# Eltrombopag inhibits the proliferation of Ewing sarcoma cells via iron chelation and impaired DNA replication

**DOI:** 10.1186/s12885-020-07668-6

**Published:** 2020-11-30

**Authors:** Torin Waters, Kelli L. Goss, Stacia L. Koppenhafer, William W. Terry, David J. Gordon

**Affiliations:** Department of Pediatrics, Division of Pediatric Hematology/Oncology, University of Iowa, 25 S Grand Avenue, Iowa City, Iowa 52242 USA

**Keywords:** Ewing sarcoma, Eltrombopag, Iron chelation

## Abstract

**Background:**

The treatment of Ewing sarcoma, an aggressive bone and soft tissue sarcoma, is associated with suboptimal outcomes and significant side-effects. Consequently, there is an urgent need to identify novel therapies that will improve outcomes for children and adults with Ewing sarcoma tumors while also decreasing treatment-related toxicities.

**Methods:**

We analyzed data from the PRISM drug repurposing screen, which tested the activity of 4518 drugs across 578 cancer cell lines, to identify drugs that selectively inhibit the growth of Ewing sarcoma cell lines. We then tested the effects of a top hit from the screen on cell proliferation, cell cycle progression, and activation of the DNA damage pathway using Ewing sarcoma cell lines. We also used a CRISPR/Cas9 gene knockout approach to investigate the role of Schlafen 11 (SLFN11), a restriction factor for DNA replication stress that is overexpressed in Ewing sarcoma tumors, in mediating the sensitivity of Ewing sarcoma cells to the drug.

**Results:**

We found that eltrombopag, an FDA-approved thrombopoietin-receptor agonist (TPO-RA) that is currently being evaluated as a treatment for chemotherapy-induced thrombocytopenia, inhibits the growth of Ewing sarcoma cell lines in vitro in proliferation and colony formation assays. However, from a mechanistic standpoint, the thrombopoietin receptor is not expressed in Ewing sarcoma cells and we show that eltrombopag impairs DNA replication and causes DNA damage in Ewing sarcoma cells by chelating iron, a known “off-target” effect of the drug. We also found that the sensitivity of Ewing sarcoma cells to eltrombopag is mediated, in part, by SLFN11, which regulates the cellular response to DNA replication stress.

**Conclusions:**

Ewing sarcoma cell lines are sensitive to eltrombopag and this drug could improve outcomes for patients with Ewing sarcoma tumors by both targeting the tumor, via chelation of iron and inhibition of DNA replication, and reducing chemotherapy-induced thrombocytopenia, via stimulation of the thrombopoietin receptor.

**Supplementary Information:**

**Supplementary information** accompanies this paper at 10.1186/s12885-020-07668-6.

## Background

Ewing sarcoma is an aggressive bone and soft tissue cancer that occurs primarily in children and young adults [1, 2]. The driver oncogene in most Ewing sarcoma tumors, EWS-FLI1, was identified over two decade ago, but directly targeting this oncogene has proven challenging [3, 4]. Consequently, the current therapy for Ewing sarcoma consists of cytotoxic chemotherapy in combination with surgery and/or radiation therapy. The AEWS0031 clinical trial (2001–2005) showed that chemotherapy intensification through interval compression, in which alternating cycles of vincristine-doxorubicin-cyclophosphamide and ifosfamide-etoposide (VDC/IE) are given every 2 weeks rather than every 3 weeks, improves the outcome for children and adults with Ewing sarcoma [5]. However, despite this treatment with intensive chemotherapy, the outcomes for children and adults with Ewing sarcoma remain suboptimal.

The VDC/IE chemotherapy regimen also causes significant on- and off-treatment toxicities, including myelosuppression, heart failure, infertility, and renal damage [5, 6]. Notably, chemotherapy-induced myelosuppression can cause significant morbidity and mortality in cancer patients. Myelosuppression can also compromise treatment outcomes, particularly in tumors where the dose-intensity of chemotherapy is critical, because it is frequently managed by delaying and/or reducing chemotherapy doses. Consequently, there is an unmet need to identify novel therapeutic approaches to treat Ewing sarcoma that will both improve outcomes and decrease treatment-related toxicities. In previous work, we used a stem cell model of Ewing sarcoma and a gene-expression based screening approach to identify that Ewing sarcoma tumors are sensitive in vitro and in vivo to iron chelators [7].

In the current study, we analyzed the Broad Institute’s PRISM drug repurposing screen, which includes 12 Ewing sarcoma cell lines, to identify drugs that selectively impair the growth of the Ewing sarcoma cell lines compared to cell lines derived from other types of cancer [8]. Eltrombopag, which is an FDA-approved thrombopoietin-receptor agonist (TPO-RA) that is currently being evaluated as a treatment for chemotherapy-induced thrombocytopenia, was a top hit in the screen [9–11]. However, the canonical target of eltrombopag, the thrombopoietin receptor (MPL), is not expressed in Ewing sarcoma cells and we show that the growth inhibitory effect of eltrombopag is due, in part, to the iron chelation properties of the drug [12–14]. Moreover, from a mechanistic standpoint, we show that eltrombopag impairs DNA replication and causes DNA damage in Ewing sarcoma cells. Overall, these results suggest that eltrombopag could have a dual role in the treatment of Ewing sarcoma tumors by both inhibiting tumor growth, via iron chelation and induction of DNA replication stress, and reducing chemotherapy-induced myelosuppression, via stimulation of the thrombopoietin receptor.

## Methods

### Cell lines and culture

Cell lines were maintained at 37^o^ C in a 5% CO_2_ atmosphere. The A673, TC32, TC71, and EW8 cell lines were provided by Dr. Kimberly Stegmaier (Dana-Farber Cancer Institute, Boston, MA). The ES6 cell line was kindly provided by the St. Jude Childhood Solid Tumor Network. The CB-AGPN cell line was obtained from the Childhood Cancer Repository (Children’s Oncology Group). The HT1080 and U2OS cell lines were obtained from ATCC. Cells were grown in Dulbecco’s Modified Eagle’s Media (DMEM) supplemented with 10% FBS, 100 IU ml^− 1^ penicillin and 100 μg ml^− 1^ streptomycin. Cell lines were used within 8–10 thawing passages and DNA fingerprinting confirmation was performed using the short tandem repeat method.

### PRISM drug screen analysis

Data obtained from the PRISM repurposing drug screen (Broad Institute, Cancer Dependency Map), which tested the activity of 4518 drugs across 578 cancer cell lines, were used to identify drugs that selectively reduce the growth of Ewing sarcoma cell lines compared to other cell lines [15]. Compound selectivity for Ewing sarcoma cell lines, compared to cell lines from other tumor types, was assessed using the log2 fold change in cell growth, *p*-value, and T-score.

### *MPL* mRNA expression

*MPL* mRNA expression data for cell lines was obtained from the Cancer Dependency Map (Broad Institute) [15]. *MPL* mRNA expression data for primary tumors was obtained from The Cancer Genome Atlas (TCGA) via cBioPortal for Cancer Genomics [16].

### Chemical compounds

Eltrombopag was obtained from MedChemExpress.

### Cell viability assay

Cell proliferation was measured using the AlamarBlue (resazurin) fluorescence assay, as previously described [17]. Approximately 5 × 10^4^ cells were plated per well of a 96-well plate, after which the cells were exposed to a range of drug concentrations for 72 h. Fluorescence readings were then obtained after adding AlamarBlue (Sigma) using a FLUOstar Omega microplate reader (BMG Labtech). IC50 values were calculated using log-transformed and normalized data (GraphPad Prism 8.3).

### Colony formation assay

A673, EW8, TC71, CB-AGPN, and U2OS cells growing in 6-well plates in triplicate were exposed to DMSO or 5 μM eltrombopag for 14 days. Crystal Violet was used to stain the colonies and the number of colonies per well were counted manually.

### Protein isolation and immunoblotting

Protein extracts for immunoblotting were prepared by incubating cells in RIPA buffer (Boston BioProducts), supplemented with protease and phosphatase inhibitors (Halt Protease & Phosphatase Inhibitor Cocktail, EDTA-free; ThermoFisher Scientific), for 20 min. Supernatants were collected after centrifugation, 17,000 r.c.f. for 15 min, at 4^o^ C. The BCA reagent (Pierce) was used to determine the protein concentrations in the samples. SDS-PAGE was used to separate proteins, which were then transferred to polyvinylidene difluoride membranes (Millipore). Antibodies to the following proteins were used in the immunoblots: phospho-Histone H2A.X (Ser139, Cell Signaling, #9718, 1:1000), phospho-CHK1 (Ser345, Cell Signaling, #2348, 1:1000), CHK1 (Cell Signaling, #2360, 1:1000), SLFN11 (Santa Cruz Biotechnology, sc-374,339), and Actin (Cell Signaling, #4970, 1:1000).

### γH2AX flow cytometry

Cells (3 × 10^5^ cells/well) were plated in a 6-well plate and allowed to adhere overnight. The cells were then treated with eltrombopag, or vehicle, for 48 h and then labeled with 5-ethynyl-2′-deoxyuridine (EdU) for 2 h. Flow cytometry for γH2AX and EdU was then performed on a Becton Dickinson LSR II instrument as described [17, 18].

### SLFN11 knockout cell lines

CRISPR/Cas9-mediated knockout of SLFN11 was performed using a lentivirus pLV plasmid (VectorBuilder) that co-expresses Cas9 and a gRNA (GAGTCCTGAGAGCAGCGCAG) targeting *SLFN11*. Lentivirus was prepared as described in previous publications and cells were selected in 1 μg/mL puromycin 48 h after transduction [17, 19, 20]. The knockout cell lines were then single-cell cloned using flow cytometry (Becton Dickinson FACS Aria).

### Cell cycle analysis

Cell-cycle analysis was performed in duplicate using the Click-iT EdU-488 Kit for flow cytometry (Thermo Fisher Scientific). Cells were labeled with EdU for 2 h and analysis was performed according to the manufacturer’s instructions. Flow cytometry was performed on a Becton Dickinson LSR II instrument.

### PIP-FUCCI cell imaging

A lentiviral (pLV) plasmid expressing the PIP-FUCCI (PCNA-interacting protein-Fluorescent Ubiquitination-based Cell Cycle Indicator) construct was obtained from VectorBuilder [21]. Lentivirus was prepared as described in previous publications and fluorescent cells were then isolated using flow cytometry (Becton Dickinson FACS Aria). Cells were imaged using a Nikon Eclipse Ti Microscope equipped with a X-cite 120Q fluorescent lamp.

### Statistical analysis

Student’s *t*-test was used to calculate *p*-values. Statistical analyses were conducted using GraphPad Prism 8.3.

## Results

### Analysis of the PRISM drug screen data identifies eltrombopag as a potent inhibitor of Ewing sarcoma cell lines

**Fig. 1:**
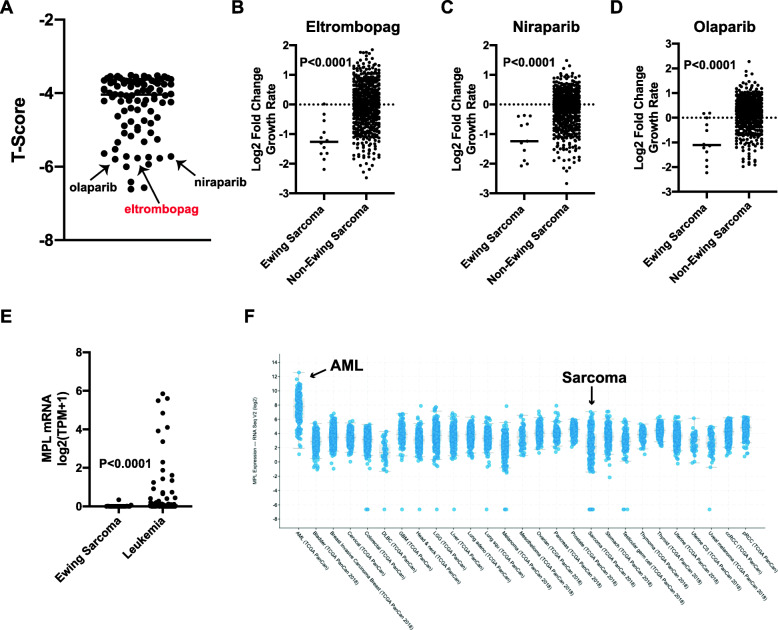
Analysis of the PRISM drug screen data identifies eltrombopag as an inhibitor of the growth of Ewing sarcoma cell lines. **a** The PRISM drug repurposing screen was analyzed to identify drug dependencies enriched in the Ewing sarcoma cell lines compared to the other cancer cell lines in the screen. Drugs with a T-score < − 3.5 are included on the plot. **b-d** Comparison of the log2 fold change in cell growth between Ewing sarcoma cells and other cancer types treated in the PRISM screen with **b** eltrombopag, **c** niraparib, and **d** olaparib. **e-f** Expression level of *MPL* mRNA in (**e**) Ewing sarcoma cell lines and (**f**) primary sarcoma tumors

The Broad Institute’s PRISM drug repurposing screen tested 4518 oncology drugs across 578 human cancer cell lines, including 12 Ewing sarcoma cell lines (Supplementary Table 1) [8]. We used this publicly-available (Cancer Dependency Map) screen data to identify drug dependencies enriched in the Ewing sarcoma cell lines compared to the other cancer cell lines in the screen (Fig. 1a and Supplementary Table 2) [15]. Notably, two of the top drug hits with selective growth inhibition for Ewing sarcoma cell lines were niraparib and olaparib, which are PARP inhibitors with well-described selectivity toward Ewing sarcoma cell lines in vitro (Fig. 1b-d) [22–30]. However, we also noted that eltrombopag (ELT), a thrombopoietin receptor (MPL) agonist, was a top hit in the screen and demonstrated similar selectivity for the Ewing sarcoma cell lines as the PARP inhibitors, which was unexpected because MPL is not expected to be expressed in sarcoma cells [9]. The thrombopoietin receptor is normally expressed on platelets, megakaryocytes, and other bone marrow cells and Fig. 1e shows that the Ewing sarcoma cell lines used in the PRISM screen do not express *MPL* mRNA [15, 31–33]. Similarly, analysis of TCGA data for primary tumors showed that *MPL* mRNA is expressed in some acute myeloid leukemias (AML), but not in sarcomas (Fig. 1f) [16]. The lack of expression of the canonical target of eltrombopag in Ewing sarcoma cells suggests that the growth inhibitory effect of the drug in the screen is “off-target,” or a false positive result.

### Eltrombopag inhibits Ewing sarcoma growth in vitro

**Fig. 2:**
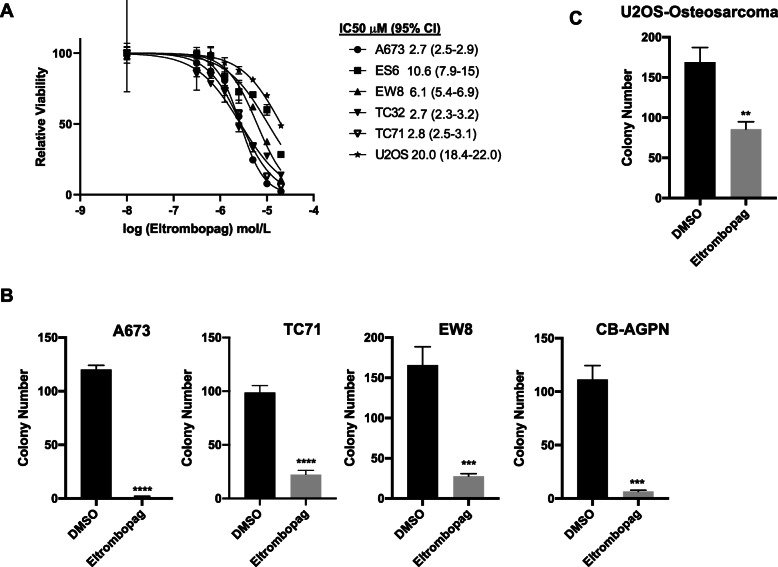
Eltrombopag inhibits Ewing sarcoma growth in vitro. **a** Dose response curves for Ewing sarcoma and osteosarcoma (U2OS) cell lines treated with different concentrations of eltrombopag. Cell viability was assessed 72 h after drug was added using the AlamarBlue assay. Error bars represent the mean ± SD of three technical replicates. **b-c** Colony formation assay for (**b**) Ewing sarcoma and (**c**) osteosarcoma cell lines treated with eltrombopag 5 μM for 14 days. Error bars represent the mean ± SD of three technical replicates. ** indicates *P* < 0.01, *** indicates *P* < 0.001, **** indicates *P* < 0.0001

Next, to validate the PRISM screen results, we performed dose-response assays with eltrombopag and five Ewing sarcoma cell lines, as well as an osteosarcoma cell line (U2OS). Figure 2a shows that eltrombopag inhibits the growth of Ewing sarcoma cell lines with an IC50 ranging between 2.7 μM and 10.6 μM, which is comparable to the serum level of the drug that is achieved in vivo in patients treated with eltrombopag [9, 10, 34, 35]. The osteosarcoma cell line U2OS was ~ 2–5-fold less sensitive to eltrombopag than the Ewing sarcoma cell lines. Eltrombopag also significantly reduced the growth of Ewing sarcoma cell lines in a colony formation assay (Fig. 2b). Again, the osteosarcoma cell line was more resistant to eltrombopag in the colony formation assay than the Ewing sarcoma cell lines (Fig. 2c). Overall, these results validate the PRISM screen data and demonstrate that eltrombopag inhibits the growth of Ewing sarcoma cells in vitro. However, as noted above, the canonical target of eltrombopag, the thrombopoietin receptor, is not expressed in Ewing sarcoma cell lines, which suggests that the mechanism of action of growth inhibition in Ewing sarcoma cells may be due to an off-target effect of the drug.

### Pretreatment of Ewing sarcoma cells with iron partially rescues the growth inhibitory effects of eltrombopag

**Fig. 3:**
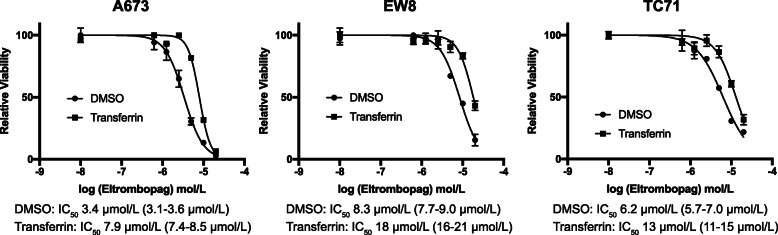
Pretreatment of Ewing sarcoma cells with iron partially rescues the growth inhibitory effects of eltrombopag. Ewing sarcoma cell lines were pre-treated with transferrin, a bioavailable form of iron, or vehicle, for 24 h. Different concentrations of eltrombopag were then added and cell viability was assessed 72 h later using the AlamarBlue assay. Error bars represent the mean ± SD of three technical replicates

Eltrombopag, in addition to its actions as a TPO-RA, also binds and chelates iron [12–14]. Notably, in prior work, we showed that Ewing sarcoma cells are uniquely sensitive to iron chelators, including ciclopirox, deferoxamine, and deferasirox, both in vitro and in vivo in xenograft experiments [7]. Consequently, we next tested whether iron mediates the toxicity of eltrombopag in Ewing sarcoma cells. Figure 3 shows that the addition of holo-transferrin, a source of biologically available iron, to the cell culture media significantly rescued the toxicity of eltrombopag toward Ewing sarcoma cells, demonstrating that iron is one target of eltrombopag in Ewing sarcoma cells.

### Eltrombopag impairs DNA replication in Ewing sarcoma cells

**Fig. 4:**
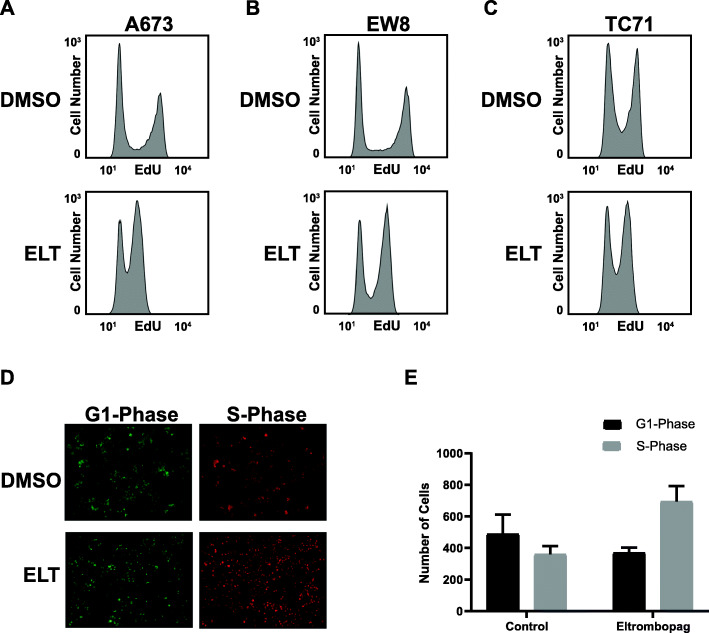
Eltrombopag impairs DNA replication in Ewing sarcoma cells. **a-c** Ewing sarcoma cell lines were treated with eltrombopag 5 μM for 18 h. Cells were then labeled with EdU and fixed for flow cytometry. **d** Representative photographs of EW8 cells expressing the PIP-FUCCI fluorescent reporter system for cell cycle phase analysis treated with vehicle or eltrombopag. **e** Quantification of PIP-FUCCI fluorescent cells, as described in (**d**). The experiment was performed in triplicate

In previous work, we identified that the iron-dependent RRM2 subunit of ribonucleotide reductase (RNR), which is the rate limiting and iron-dependent enzyme in the synthesis of deoxyribonucleotides and required for DNA replication, is one target of iron chelators in Ewing sarcoma cells [7, 17, 20]. Inhibition of RNR is known to deplete deoxyribonucleotides, impair DNA replication, cause DNA replication stress, and activate the ataxia telangiectasia and Rad3-related protein (ATR)-checkpoint kinase 1 (CHK1) DNA damage response pathway [17, 19, 20, 36–39]. Figure 4a-c show that eltrombopag impairs DNA replication in Ewing sarcoma cell lines, as assessed using an EdU incorporation assay. Next, we generated an EW8 Ewing sarcoma cell line that expresses the “PIP-FUCCI” fluorescent reporter system, which identifies cell cycle phases in live cells using fluorescent marker proteins [21]. Figure 4d-e show that eltrombopag significantly increases the number of cells in S-phase, consistent with impaired DNA replication.

### Eltrombopag causes DNA damage and activates the CHK1 pathway in Ewing sarcoma cells

**Fig. 5:**
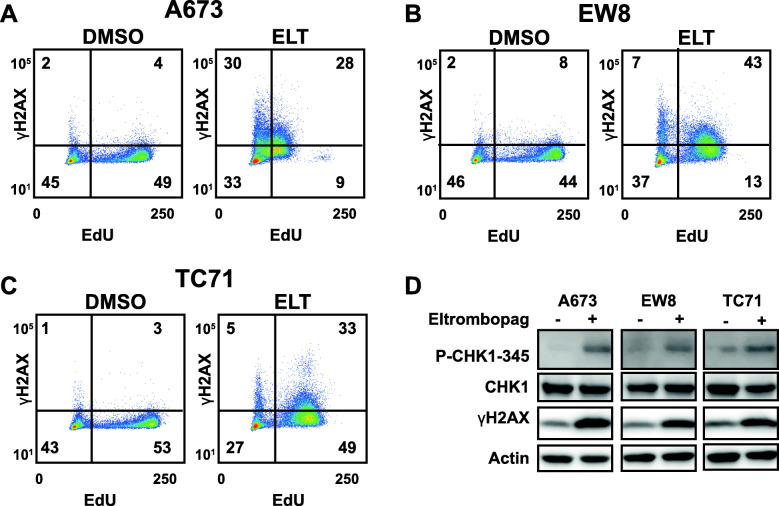
Eltrombopag causes DNA damage and activates the CHK1 pathway in Ewing sarcoma cells. **a-c** Ewing sarcoma cell lines were treated with eltrombopag 5 μM or vehicle for 18 h. Cells were then labeled with EdU and fixed for flow cytometry for γH2AX, a marker of DNA damage. **d** Ewing sarcoma cell lines were treated with eltrombopag as described in (**a**) and then cellular lysates were collected for immunoblotting. The blots have been cropped and full-length blots are presented in Supplementary Figure 2

DNA replication stress, due to inhibition of RRM2 or other causes, generates DNA damage and activates the CHK1 DNA damage response pathway [20, 37–39]. Consequently, we treated Ewing sarcoma cell lines with eltrombopag, labeled cells with EdU to identify cells in S-phase, and then stained the cells for the marker of DNA damage γH2AX [18, 40–42]. Figure 5a-c demonstrate that eltrombopag causes DNA damage in cells in S-phase (EdU positive cells). Furthermore, eltrombopag treatment also activates the ATR-CHK1 DNA damage response pathway in Ewing sarcoma cells, as assessed by the phosphorylation of CHK1 at Ser345 (Fig. 5d). Treatment of cells with a caspase inhibitor, Z-VAD-FMK, did not block the phosphorylation H2AX caused by eltrombopag, suggesting that the DNA damage is not secondary to apoptosis (Supplementary Figure 1).

### SLFN11 mediates toxicity to eltrombopag in Ewing sarcoma cells

**Fig. 6:**
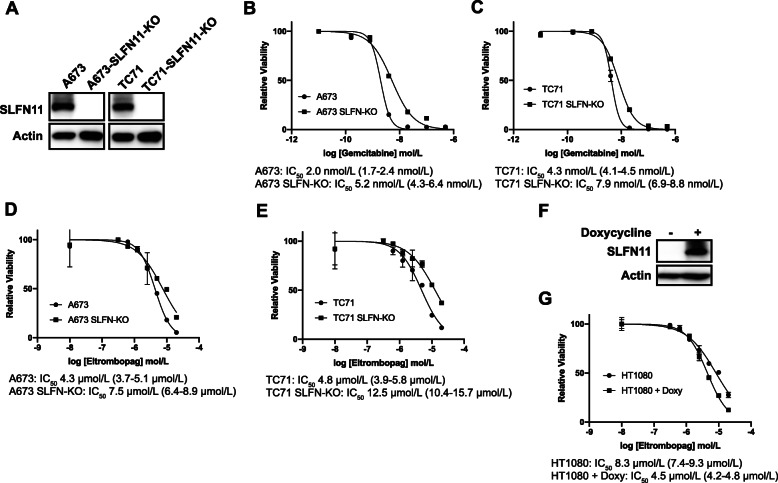
SLFN11 mediates toxicity to eltrombopag in Ewing sarcoma cells. **a** Immunoblot for SLFN11 after CRISPR/Cas9-mediated gene knockout. The blots have been cropped and full-length blots are presented in Supplementary Figure 2. **b-c** Dose response curves for SLFN11-KO and parental cell lines treated with different concentrations of gemcitabine. Cell viability was assessed 72 h after drug was added using the AlamarBlue assay. Error bars represent the mean ± SD of three technical replicates. **d-e** Dose response curves for SLFN11-KO and parental cell lines treated with different concentrations of eltrombopag. Cell viability was assessed 72 h after drug was added using the AlamarBlue assay. Error bars represent the mean ± SD of three technical replicates. **f** HT1080 cells with doxycycline-inducible SLFN11 were treated with doxycycline for 24 h. Cellular lysates were then collected for immunoblotting. **g** The HT1080-SLFN11 cells, grown in the presence of doxycycline or vehicle, were treated with different concentrations of eltrombopag. Cell viability was assessed 72 h after drug was added using the AlamarBlue assay. Error bars represent the mean ± SD of three technical replicates. The results are representative of two independent experiments

SLFN11 is overexpressed in Ewing sarcoma tumors and is known to sensitize cells to a range of drugs that cause DNA replication stress, including iron chelators [7, 43–48]. Consequently, we used CRISPR/Cas9 to knockout SLFN11 in two Ewing sarcoma cell lines, A673 and TC71 (Fig. 6a). As a control, we first treated the SLFN11 knockout and parental cells with gemcitabine, which is a drug that inhibits the RRM1 subunit of RNR and causes DNA replication stress. SLFN11 has also been reported to mediate the sensitivity of cells to gemcitabine [46]. Figure 6b shows that the knockout of SLFN11 decreases the sensitivity of Ewing sarcoma cells to gemcitabine by 2-fold, based on IC50 measurements. Similarly, the SLFN11 knockout cell lines are also ~ 2-fold more resistant to eltrombopag than the parental cell lines. Next, we used a doxycycline-inducible system to express SLFN11 in a fibrosarcoma cell line, HT1080, that does not normally express SLFN11 (Fig. 6f) [49]. Figure 6g shows that the expression of SLFN11 in the HT1080 cells increased the sensitivity of the cells to eltrombopag by 1.8-fold. Overall, these results demonstrate that the high level of SLFN11 expressed in Ewing sarcoma cells mediates, in part, the enhanced sensitivity to eltrombopag.

## Discussion

The standard therapy for Ewing sarcoma, which consists of alternating cycles of vincristine-doxorubicin-cyclophosphamide and ifosfamide-etoposide (VDC/IE) administered on an interval-compressed schedule, is associated with overall survival rates of ~ 20% and ~ 70% for patients with metastatic and localized tumors, respectively [1, 2, 5]. This intensive chemotherapy regimen also causes significant on- and off-treatment toxicities, including myelosuppression [5, 6]. Notably, reducing the impact of treatment-related side-effects is a central focus of clinical trials for other pediatric cancers, such as acute lymphoblastic leukemia, that have higher rates of survival [50]. Given the suboptimal survival rates for Ewing sarcoma patients, a reduction in therapy intensity is not feasible at this time. However, the addition of well-tolerated agents that have the potential to mitigate current treatment-related morbidities, while also contributing to tumor toxicity, is a promising alternative approach.

Eltrombopag and other thrombopoietin-receptor agonists are currently under investigation as potential supportive care agents to help reduce the duration and severity of thrombocytopenia in patients receiving myelosuppressive chemotherapy, in a similar fashion as to how granulocyte-colony stimulating factor (G-CSF) is used to help reduce the time to neutrophil recovery [10, 11, 51]. While the data are not yet available to recommend the routine use of eltrombopag in combination with myelosuppressive chemotherapy, eltrombopag is approved for use in pediatric patients with chronic immune thrombocytopenic purpura (ITP) and aplastic anemia [52]. Eltrombopag is also known to have a tolerable side-effect profile in both children and adults [53, 54].

In this work, we used data available through the PRISM drug repurposing screen to identify that eltrombopag selectively reduces the growth of Ewing sarcoma cell lines compared to cell lines from other tumor types [8, 15]. We then showed that eltrombopag, used at clinically relevant doses, reduces the growth of Ewing sarcoma cells in vitro in proliferation and colony formation assays. In addition, from a mechanistic standpoint, eltrombopag impairs DNA replication, causes DNA damage, and activates the CHK1 DNA damage response pathway. The canonical target of eltrombopag, the thrombopoietin receptor (MPL), is not expressed in Ewing sarcoma tumors. Instead, we identified that the growth inhibitory effect of eltrombopag is due, in part, to the iron chelation properties of the drug, which provides further support for our previous work that identified iron chelator drugs as a unique vulnerability of Ewing sarcoma tumor in vitro and in vivo [7]. Notably, eltrombopag has also been reported to target additional cancer types, including leukemia and hepatocellular carcinoma, via an iron chelation mechanism [12, 13, 55].

Identification of this off-target mechanism of eltrombopag in inhibiting the growth of Ewing sarcoma cells could aid in optimizing the anti-cancer efficacy of the drug. For example, we showed that eltrombopag activates the CHK1 DNA damage response pathway. Combining eltrombopag with CHK1 inhibitors, which are being tested in early phase clinical trials, could generate synergistic toxicity [17, 38, 39]. Based on our previous work with the iron chelator ciclopirox, which only had a modest effect as a single agent in reducing the growth of a Ewing sarcoma xenograft in vivo, we expect that future work will need to focus on identifying drug combination strategies [7]. Drug synergy testing could be used to find an optimal drug combination, but adding eltrombopag to a regimen with a significant incidence of thrombocytopenia may be more clinically relevant. For example, thrombocytopenia is a common side effect of the standard, first-line chemotherapy regimen (VDC/IE) used to treat Ewing sarcoma [5, 56]. In addition, we identified that the overexpression of SLFN11 in Ewing sarcoma tumors mediates sensitivity to eltrombopag, which provides an explanation for some of the specificity of eltrombopag for Ewing sarcoma tumors in the screen. Furthermore, these data suggest that SLFN11 expression could be used as a biomarker to identify Ewing sarcoma tumors with sensitivity to eltrombopag, as well as identify other cancer types that could benefit from eltrombopag treatment [44, 46, 57, 58].

## Conclusions

In conclusion, our data suggest that eltrombopag, a well-tolerated drug used to treat pediatric chronic ITP and aplastic anemia, could provide therapeutic benefit for patients with Ewing sarcoma by both mitigating treatment-related thrombocytopenia and contributing additional anti-tumor toxicity via iron chelation and RNR inhibition.

## Supplementary Information

**Additional file 1: Supplementary Figure 1.** Ewing sarcoma cell lines were treated with vehicle, eltrombopag 5 μM, Z-VAD-FMK 10 μM, or the combination of eltrombopag and Z-VAD-FMK for 18 h and then cellular lysates were collected for immunoblotting. The blots have been cropped and full-length blots are presented in Supplementary Figure 2.

**Additional file 2: Supplementary Figure 2.** Full-length blots for Figs. 5d, 6a, 6f, and Supplementary Figure 1.

**Additional file 3: Supplementary Table 1.** Ewing sarcoma cell lines included in the PRISM drug screen.

**Additional file 4: Supplementary Table 2.** Drug dependencies enriched in the Ewing sarcoma cell lines.

## Data Availability

All data generated or analyzed during this study are included in this published article and its supplementary information files. The PRISM drug screening datasets analyzed in the current study are publicly available through the Cancer Dependency Map (depmap.org).

## Abbreviations

CRISPR: Clustered regularly interspaced short palindromic repeats
TPO-RA: Thrombopoietin-receptor agonist
VDC/IE: Vincristine-doxorubicin-cyclophosphamide and ifosfamide-etoposide
EdU: 5-ethynyl-2′-deoxyuridine
PIP-FUCCI: PCNA-interacting protein-Fluorescent Ubiquitination-based Cell Cycle Indicator
MPL: MPL proto-oncogene, thrombopoietin receptor
RNR: Ribonucleotide reductase
RRM2: Ribonucleotide reductase M2
ATR: Ataxia telangiectasia and Rad3-related protein
CHK1: Checkpoint kinase 1
G-CSF: Granulocyte-colony stimulating factor
ITP: Immune thrombocytopenic purpura
