# Supplementary figures and images for: Eltrombopag inhibits the proliferation of Ewing sarcoma cells via iron chelation and impaired DNA replication
Source: BMC Cancer. 2020 Nov 30;20:1171. doi: 10.1186/s12885-020-07668-6 (PMC7706234; doi:10.1186/s12885-020-07668-6)

Supplementary Figure 1

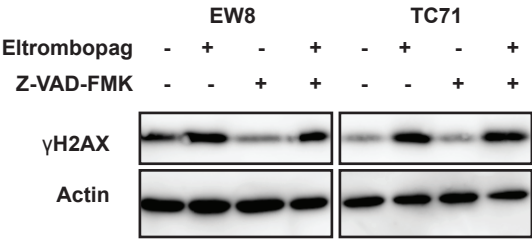

Supplement: Supplementary file 1 — Additional file 1: Supplementary Figure 1. Ewing sarcoma cell lines were treated with vehicle, eltrombopag 5 μM, Z-VAD-FMK 10 μM, or the combination of eltrombopag and Z-VAD-FMK for 18 h and then cellular lysates were collected for immunoblotting. The blots have been cropped and full-length blots are presented in Supplementary Figure 2. [file 12885_2020_7668_MOESM1_ESM.pdf]

Supplementary Figure 2

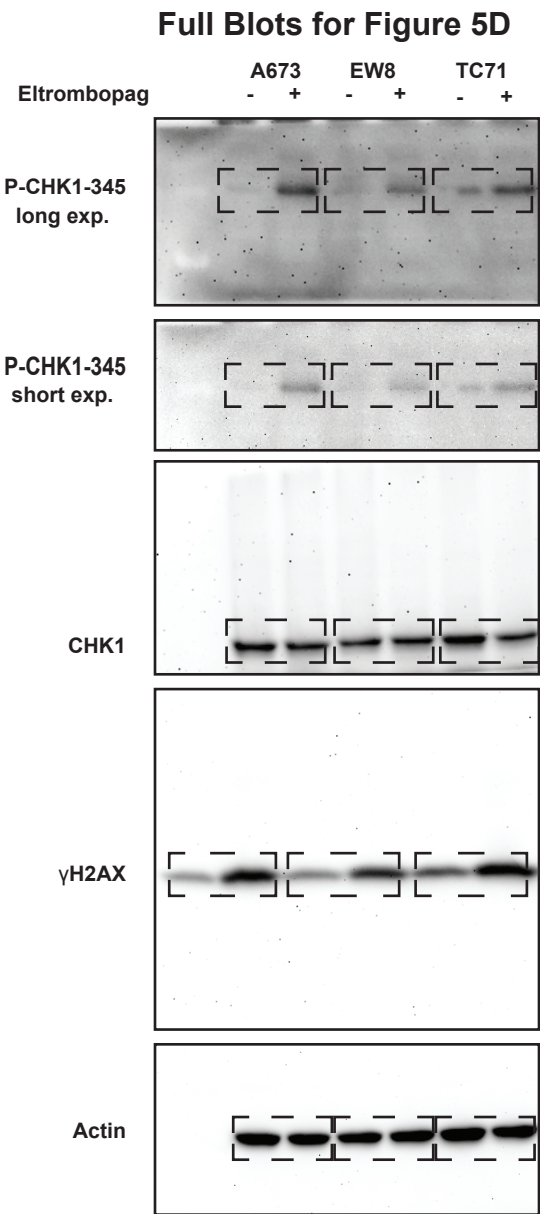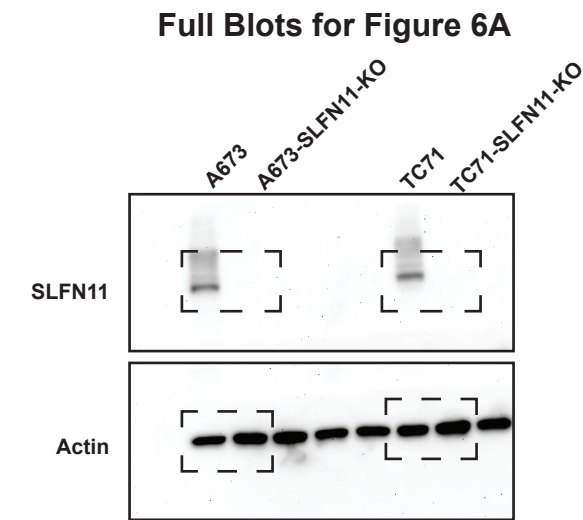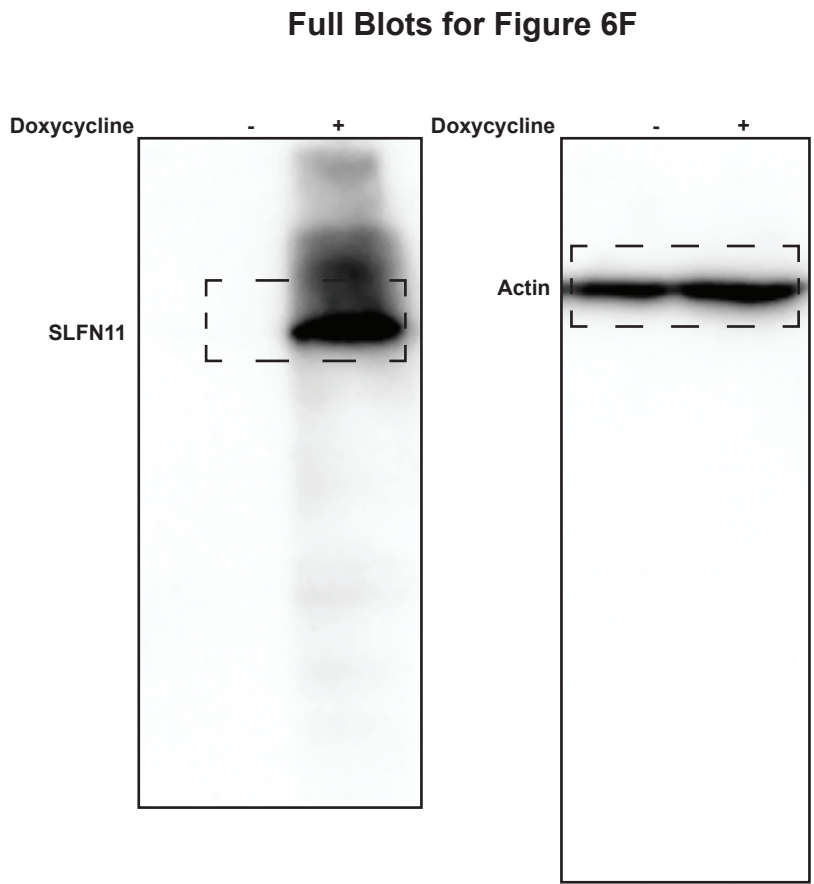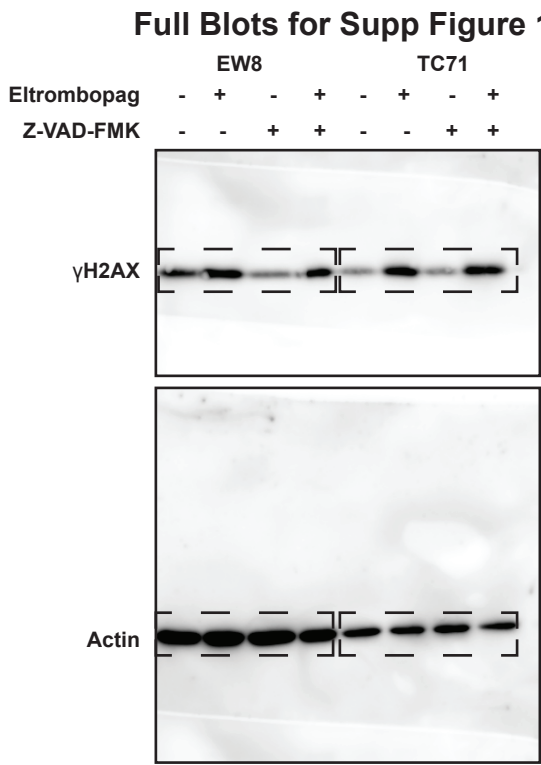

Supplement: Supplementary file 2 — Additional file 2: Supplementary Figure 2. Full-length blots for Figs. 5d, 6a, 6f, and Supplementary Figure 1. [file 12885_2020_7668_MOESM2_ESM.pdf]
